# Supplementary material for: The Systems Biology Research Tool: evolvable open-source software
Source: BMC Syst Biol. 2008 Jun 29;2:55. doi: 10.1186/1752-0509-2-55 (PMC2446383; doi:10.1186/1752-0509-2-55)
Supplement: Additional file 1 — SBRT Archive. An archive of the current version of the Systems Biology Research Tool. [file 1752-0509-2-55-S1.zip › sbrt-1.4.0/doc/developers_guide/api/sbrt/kernel/fba/comp/InitialPointComputer.Results.html]

InitialPointComputer.Results


|  |  |  |
| --- | --- | --- |
| **Overview** \| **Package** \| **Class** \| **Use** \| **Tree** \| **Deprecated** \| **Index** \| **Help** | |  |
| **PREV CLASS**   **NEXT CLASS** | **FRAMES**    **NO FRAMES**     **All Classes** |  |
| SUMMARY: NESTED \| FIELD \| CONSTR \| METHOD | DETAIL: FIELD \| CONSTR \| METHOD |  |


---


## sbrt.kernel.fba.comp Class InitialPointComputer.Results

```
java.lang.Object
  sbrt.kernel.fba.comp.InitialPointComputer.Results
```

**Enclosing class:**: InitialPointComputer

---

``` public class InitialPointComputer.Results extends java.lang.Object ```

This class is used to represent the results of a

**Author:**
:   wright

---

| **Method Summary** | |
| --- | --- |
| `double` | `getCentrality()`             Returns the computed measure of centrality of the flux vector. |
| `java.util.Map<java.lang.String,java.lang.Double>` | `getCentralityVector()`             Returns the centrality vector. |
| `java.util.Map<java.lang.String,java.lang.Double>` | `getFluxVector()`             Returns the computed flux vector. |

**Methods inherited from class java.lang.Object**

`clone, equals, finalize, getClass, hashCode, notify, notifyAll, toString, wait, wait, wait`

**Method Detail**

### getCentrality

```
public double getCentrality()
```

:   Returns the computed measure of centrality of the flux vector.

    :   **Returns:**: the computed measure of centrality of the flux vector.

---


### getFluxVector

```
public java.util.Map<java.lang.String,java.lang.Double> getFluxVector()
```

:   Returns the computed flux vector.

    :   **Returns:**: the computed flux vector.

---


### getCentralityVector

```
public java.util.Map<java.lang.String,java.lang.Double> getCentralityVector()
```

:   Returns the centrality vector.

    :   **Returns:**: reaction names mapped to their centrality value.


---


|  |  |  |
| --- | --- | --- |
| **Overview** \| **Package** \| **Class** \| **Use** \| **Tree** \| **Deprecated** \| **Index** \| **Help** | |  |
| **PREV CLASS**   **NEXT CLASS** | **FRAMES**    **NO FRAMES**     **All Classes** |  |
| SUMMARY: NESTED \| FIELD \| CONSTR \| METHOD | DETAIL: FIELD \| CONSTR \| METHOD |  |


---
